# Infancy and Infant-Rearing

**Published:** 1895-12

**Authors:** 


*Infancy and Infant-Rearing.* By JOHN BENJAMIN HELLIER, M.D. Pp. x., 121. London: CHARLES GRIFFIN & COMPANY, LIMITED. 1895.

Dr. Hellier has brought out a thoroughly readable book in this small volume; but has attempted too much in trying to suit at the same time the requirements of the nurse, medical student, and practitioner. From considerable experience gained in reviewing manuals of this kind, we believe that it is impossible for an author to express himself in sufficient detail both to satisfy the practitioner and at the same time, by avoiding the use of scientific terms, to make his book suitable for a nurse. Practitioners should find nothing to complain of in this work, but we can well imagine a nurse finding it too full of words of which she does not know the full meaning. The style is good and clear, and the author's views are well expressed. Some of the diagrams and tables are new, but we recognise a few old friends. The index is full and correct.

